# Complete genome sequence of *Mannheimia bovis* isolated from a healthy feedlot calf in Saskatchewan, Canada

**DOI:** 10.1128/MRA.00456-23

**Published:** 2023-08-09

**Authors:** Darien Deschner, Dhinesh Periyasamy, Cheryl L. Waldner, Champika Fernando, Janet E. Hill

**Affiliations:** 1 Department of Veterinary Microbiology, Western College of Veterinary Medicine, University of Saskatchewan, Saskatoon, Saskatchewan, Canada; 2 Prairie Diagnostic Services, Inc., Saskatoon, Saskatchewan, Canada; 3 Department of Large Animal Clinical Sciences, Western College of Veterinary Medicine, University of Saskatchewan, Saskatoon, Saskatchewan, Canada; University of Maryland School of Medicine, Baltimore, Maryland, USA

**Keywords:** *Mannheimia haemolytica*, cattle, genomes, Mannheimia bovis

## Abstract

A lack of whole genome sequences for *Mannheimia* spp. other than *Mannheimia haemolytica* complicates their identification. Here, we present the genome sequence of *Mannheimia bovis* 39324.S-11, isolated from a healthy calf on a feedlot in Saskatchewan, Canada, and compare it to ZY190616^T^, which is currently the only other isolate of *M. bovis* for which sequence is publicly available.

## ANNOUNCEMENT

*Mannheimia haemolytica* is a pathogen of the bovine respiratory tract that contributes to bovine respiratory disease (1, 2). Other *Mannheimia* spp. isolated from the bovine respiratory tract to date include *Mannheimia bovis*, *Mannheimia granulomatis*, and *Mannheimia varigena*; however, few isolates of these species have been characterized, so genomic and phenotypic data for species aside from *M. haemolytica* remain sparse. This lack of information can lead to misidentification in the diagnostic laboratory. The genome sequence reported here is from an isolate initially identified as *M. haemolytica* based on colony morphology and matrix-assisted laser desorption ionization–time of flight (MALDI-TOF) mass spectrometry analysis (MALDI Biotyper, Bruker Daltonics). *M. bovis* was recently designated as a new species based on a single isolate (ZY190616^T^) from the lung of a dead cow (3). The genome sequence of ZY190616^T^ is published, but this species is not represented in the MALDI Biotyper reference library. The genome sequence of 39324.S-11 (CP125805) is reported to provide more data on this species, improving the identification of *Mannheimia* spp.

Isolate 39324.S-11 was isolated on chocolate agar (35°C, 5% CO_2_, and 48 h) from a nasopharyngeal swab from a healthy calf on a Saskatchewan feedlot in 2021 (4). Genomic DNA was extracted from a broth culture (brain heart infusion + 1% glucose, 37°C) using a salting-out protocol (5) and used for both Illumina and Nanopore sequencing. Illumina sequencing libraries were prepared using the Nextera XT Library Prep Kit (Illumina). DNA was sequenced along with 19 other genomes using the 500-cycle MiSeq Reagent Kit v2 on an Illumina MiSeq. Long-read data were generated on a GridION using the ligation sequencing kit (SQK-LSK109) and the FLO-MIN106 flow cell with MinKnow v 22.03.2 OS (Oxford Nanopore Technologies) and Guppy v 6.06 basecaller (ONT) with the high accuracy basecalling option. No shearing or size selection was performed during library preparation. A *Q*-score cutoff of 9 was used for the GridION run. Illumina reads were processed using Trimmomatic v 0.39 with a sliding window of 4, *Q*-score threshold of 20, and minimum sequence length of 36 bp (6). Nanopore reads were processed using filtlong v 0.2.1 with a minimum sequence length of 1,000 bp (7). The read N50 for Nanopore reads was 19,394 bp [NanoPlot (8)]. Quality-filtered Illumina data (263,891,180 bp) and Nanopore data (180,047,140 bp) were used for *de novo* whole genome assembly with Unicycler v 0.5.0, and the resulting single contig (197× coverage) was circularized by Unicycler prior to annotation with the NCBI Prokaryotic Genome Annotation Pipeline v 6.5 (9 - 12). Default parameters were used for all software unless otherwise specified.

The resulting genome is 2,249,793 bp with a GC content of 39.7% and 2,090 protein coding sequences. Comparison of this sequence to the type of strain of *M. bovis* (CP061280) revealed a 16S rRNA sequence identity of 97.1%, cpn60 identity of 98.1%, and a whole genome average nucleotide identity (ANI) of 93.3% (Table 1). The relatively low ANI value is likely due to the presence of insertion of 7,761 bp in 39324.S-11, which was identified as a likely prophage based on PHASTER analysis (13).

**TABLE 1:** Assembly statistics of *Mannheimia bovis* whole-genome sequences

Isolate name	GC (%)	No. coding sequences	Size (bp)	rRNA copy numbers (5S, 16S, and 23S)	Accession number
39324.S-11	39.7	2,090	2,249,793	8, 6, and 6	CP125805
ZY190616^T^	39.8	2,047	2,155,161	8, 6, and 6	CP061280

## Data Availability

This whole-genome sequence has been deposited in NCBI GenBank with the accession number CP125805, BioProject Accession number PRJNA971657. The Illumina and Nanopore sequence reads are deposited in the sequence read archive (SRA) under the accession numbers SRR24529684 and SRR24529683, respectively.
